# Time Course of MERS-CoV Infection and Immunity in Dromedary Camels

**DOI:** 10.3201/eid2212.160382

**Published:** 2016-12

**Authors:** Benjamin Meyer, Judit Juhasz, Rajib Barua, Aungshuman Das Gupta, Fatima Hakimuddin, Victor M. Corman, Marcel A. Müller, Ulrich Wernery, Christian Drosten, Peter Nagy

**Affiliations:** University of Bonn Medical Centre, Bonn, Germany (B. Meyer, V.M. Corman, M.A. Müller, C. Drosten);; Deutsches Zentrum für Infektionsforschung, Braunschweig, Germany (V.M. Corman, C. Drosten);; Emirates Industries for Camel Milk and Products, Dubai, United Arab Emirates (J. Juhasz, R. Barua, A. Das Gupta, P. Nagy);; Central Veterinary Research Laboratory, Dubai (F. Hakimuddin, U. Wernery)

**Keywords:** MERS-CoV, Middle East respiratory syndrome coronavirus, maternal antibodies, immunity, infection, camel, dams, calves, dromedary, immunity, infection, risk assessment, viruses

## Abstract

Knowledge about immunity to Middle East respiratory syndrome coronavirus (MERS-CoV) in dromedary camels is essential for infection control and vaccination. A longitudinal study of 11 dam–calf pairs showed that calves lose maternal MERS-CoV antibodies 5–6 months postparturition and are left susceptible to infection, indicating a short window of opportunity for vaccination.

In 2012, Middle East respiratory syndrome coronavirus (MERS-CoV) emerged on the Arabian Peninsula (*1*). As of February 2016 the virus has caused 1,638 human infections, including 587 deaths (*2*). Zoonotic transmission of MERS-CoV was suspected early on (*3*). Dromedaries on the Arabian Peninsula and on the African continent have harbored MERS-CoV–specific antibodies for at least 20–30 years (*3*–*7*), long before the first human infections were recognized. Additionally, detection of MERS-CoV nucleotide sequences in throat swab specimens from camels confirmed the presence of the virus in these animals (*8*). The existence of an active animal reservoir receives additional support by epidemiologic investigations that found no sustained human-to-human transmission in MERS-CoV–affected countries such as Saudi Arabia (*9*). 

As documented, primary infections in humans have occurred through contact with infected dromedaries, and measures to prevent primary human infections need to focus on the camel-human interface (*8*,*10*). However, it has been unclear how MERS-CoV transmission is maintained in camels and which factors drive virus transmission from camels to humans. Clarifying the infection pattern of MERS-CoV in herds of dromedary camels is key to the design of herd management and vaccination strategies to control the source of human infections. Preliminary information is limited to observations of lower seroprevalences in juvenile compared with adult camels and higher viral load upon MERS-CoV infection in juveniles (*4*,*5*,*11*). Hence, infections in juvenile camels might drive transmission of MERS-CoV to humans.

## The Study

We monitored MERS-CoV–specific antibody levels in 11 pairs of camel dams and their calves at monthly intervals over the course of 1 year postparturition. These animals were born and raised on a closed commercial camel dairy farm in the United Arab Emirates that had a strict animal health and biosecurity program. The total number of camels on the farm was ≈4,500. Animals are kept in open paddocks and are grouped according to the age of their calves and production stage. Despite high standards of hygienic husbandry and biosecurity, the transmission of pathogens within the farm cannot be completely eliminated. Therefore, the entire farm is 1 epidemiologic unit. The 11 dam–calf pairs investigated in this study were kept in different fenced compartments within 100–150 m from each other. However, all these animals were kept together with other dam–calf pairs in the same paddock throughout lactation. All camel calves were born during June 3–15, 2014. 

Nasal swab specimens were taken from all 11 mothers and calves, and serum samples were obtained through jugular vein puncture. Blood cells were removed immediately after collection, and samples were stored at −20°C until testing. Serum samples and nasal swabs were taken at the day of parturition, at 1 week and 1 month postparturition, and then at monthly intervals until June 2015. MERS-CoV RNA was detected through amplification of gene targets as described previously (*12*). Virus isolation was performed on Vero cells. MERS-CoV–specific IgG and neutralizing antibodies were determined by ELISA and microneutralization test as described previously (*5*,*10*).

In general, maternal IgG antibodies in camels are not acquired via the transplacental route but through the intake of colostrum during the first 24 hours postparturition (*13*). After 24 hours, antibody levels in the dam’s milk decrease rapidly, and IgG levels in calves’ serum cease to rise (*13*). This pattern is reflected for MERS-CoV in this study. On the day of parturition, samples were collected from 5 of 11 dam–calf pairs studied. High levels of MERS-CoV–specific antibodies were observed in all dams, whereas no antibodies were detected in calves (Figure, panel A). At day 7 postparturition, however, all 11 camel calves had high MERS-CoV–specific antibody levels. These levels declined during the first 6 months postparturition, whereas IgG in dams remained constantly at high levels. The neutralizing activity of IgG was confirmed by microneutralization tests on serum samples from calves collected 2–3 months, 5–6 months, and 1 year after birth (Figure, panel B). After 5–6 months, serum from 6 of 11 calves had completely lost their neutralizing activity. The remaining 5 calves had low neutralizing titers, ranging from 1:20 to 1:40 (Figure, panel B).

**Figure:**
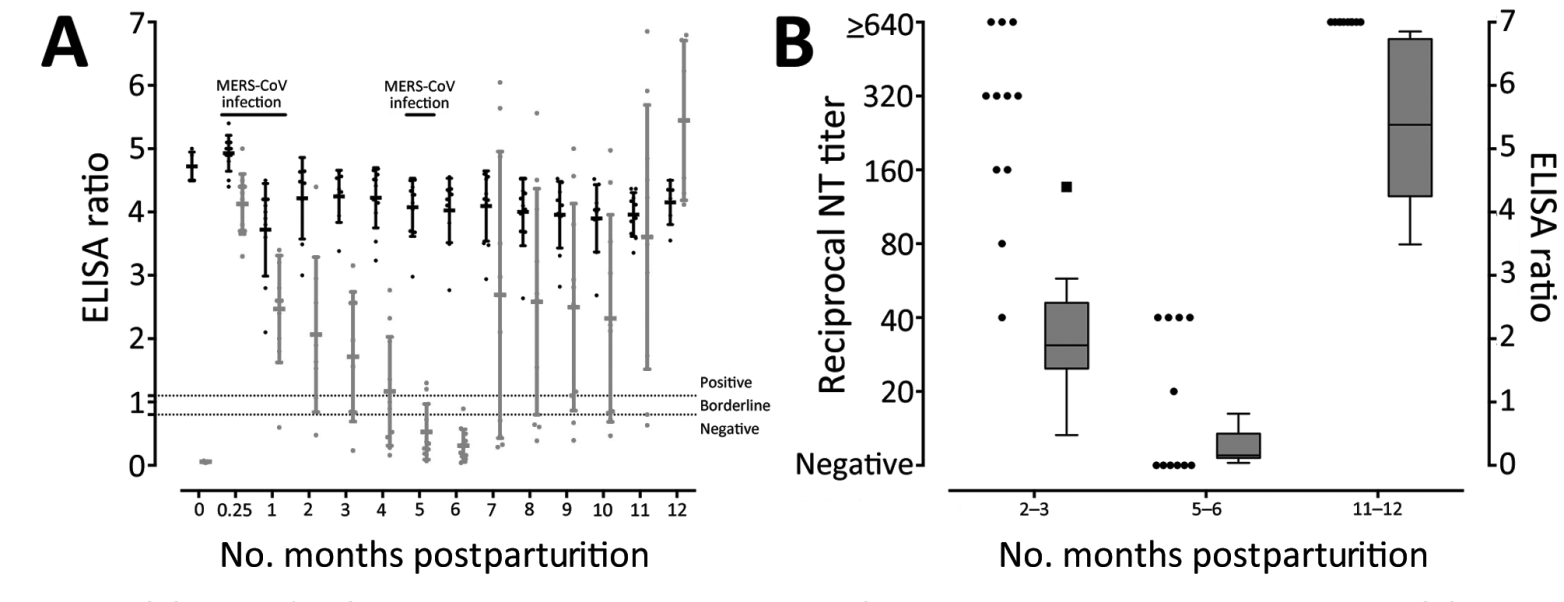
MERS-CoV–specific IgG antibody levels in dromedary camel dam–calf pairs, United Arab Emirates, 2014–2015. A) MERS-CoV spike protein S1-domain–based ELISA ratios of individual samples (dots) plus mean (horizontal line) and SD (error bars) over the course of 1 year for dams (black dots) and calves (gray dots). Ratios were calculated by dividing the ELISA optical density at 450 nm of each sample by that of a calibrator to minimize interassay variation. Dashed lines indicate cutoff values for positive (ratio 1.1) and borderline (ratio 0.8) samples. MERS-CoV infection indicates time points where MERS-CoV RNA was detected in camels. B) Neutralizing titers of individual samples from camel calves at selected time points determined by microneutralization test (dots). For comparison, ELISA ratios for the selected time points are shown in parallel as a boxplot diagram; box represents 50% of the complete dataset from the first to the third quartile, and whiskers are drawn according to the Tukey method. MERS-CoV, Middle East respiratory syndrome coronavirus; NT, neutralization test.

To examine possible correlations between antibodies and MERS-CoV infection, we examined shedding of MERS-CoV in nasal swab specimens. During the first 5 months postparturition, MERS-CoV RNA was detected sporadically on days 7 and 30 (Table). Infectious virus was isolated only from calves but not dams. At 6 months postparturition, when calves showed the lowest antibody titers, MERS-CoV RNA was detected in 2 of 11 calves (Table), indicating active MERS-CoV infection. All calves seroconverted during the following weeks (Figure, panel A); 1 calf had meanwhile been euthanized because of a congenital forelimb deformity. All calves showed high reciprocal neutralizing titers of >640 at 11–12 months postparturition.

**Table:** MERS-CoV nucleic acid detection in nasal swab specimens from camel dam–calf pairs, United Arab Emirates, 2014–2015*

Months postparturition	No. positive/no. tested (%)	
	Adults†	Calves‡
0	0/5	0/5
0.25	3/11 (27.2)	2/11 (18.2)§
1	2/11 (18.2)	5/11 (45.5)
2	0/11	0/11
3	0/11	0/11
4	0/5	0/5
5	0/11	0/11
6	0/11	2/11 (18.2)¶
7	0/10	0/10
8	0/10	0/10
9	0/10	0/10
10	0/10	0/10
11	0/10	0/10
12	0/10	0/10

*One dam–calf pair was excluded after month 6 because the calf was euthanized for an unrelated condition. MERS-CoV, Middle East respiratory syndrome coronavirus. †5 MERS-CoV–positive specimens from adult camels were from 4 individual animals. ‡9 MERS-CoV–positive specimens from camel calves were from 6 individual animals. §Virus was isolated from both infected calves. ¶Virus was isolated from 1 infected calf.

## Conclusions

This longitudinal study of natural MERS-CoV infections in camels confirms assumptions from preliminary cross-sectional studies in camels (*4*,*5*,*11*). MERS-CoV infection appears to predominantly affect young, immunologically naive animals. Serum antibodies might not have been sufficient to mediate protective immunity in the respiratory tract because dams and calves were sporadically infected even as maternal antibodies peaked at day 7 postparturition. These findings are consistent with earlier reports of MERS-CoV reinfection in seropositive camels (*4*,*11*). Nevertheless, our findings of virus isolation from calves but not dams are in line with earlier observations of reduced viral load in seropositive camels on reinfection (*11*,*14*), indicating that neutralizing antibodies might not provide sterile immunity but could still reduce the viral replication level. The predominance of infection in young animals is better explained by the absence of immunity than by other factors, such as social group density, because the number of newborn camels in our study was negligible compared with the overall size of the herd at the farm. Moreover, young camels were not kept in a contiguous group but in small compartments, where they had more contact with their mothers than with other young animals. Calves are likely to have been infected through fomites or through adult animals shedding low quantities of virus.

Our findings have important implications for the prevention of human infections through camel herd management and camel vaccination. Camel breeding, even if involving a small number of newborn animals, should be classified as a risk for human acquisition of MERS-CoV. The greatest risk should be assumed for the time after the fourth month of life until the first wave of natural infections, which should occur during the first year of life in camels raised in MERS-CoV–endemic regions. Measures for the prevention of infection, such as personal protective equipment, hand hygiene, and environmental sanitation, as applied on the farm in our study, should be sufficient for protection, given that no human MERS-CoV illnesses occurred among staff and only 2 of 300 workers with regular contact with camels had detectable MERS-CoV–specific IgG antibodies. Because persons with underlying disease and the elderly show the most severe outcomes of MERS-CoV infection, these groups should generally avoid farms where camel calves are being raised. 

Our results also suggest that studies dealing with application and efficacy of MERS-CoV vaccines should be modified. A first study involving immunologically naive animals showed a sharp decline in virus secretion after vaccination (*14*). However, future vaccination trials should also investigate the effect of preexisting maternal antibodies on vaccine efficacy (*15*).
